# Pharmacological Inhibition of PTEN Aggravates Acute Kidney Injury

**DOI:** 10.1038/s41598-017-10336-8

**Published:** 2017-08-25

**Authors:** Jun Zhou, Li Jia, Zhaoyong Hu, Yanlin Wang

**Affiliations:** 1 Selzman Institute for Kidney Health and Section of Nephrology, Department of Medicine, Baylor College of Medicine, Houston, Texas USA; 2 Department of Anesthesiology, Affiliated Foshan Hospital of Sun Yat-Sen University, Foshan, China; 3 Center for Translational Research on Inflammatory Diseases (CTRID) and Renal Section, Michael E. DeBakey Veterans Affairs Medical Center, Houston, Texas USA

## Abstract

Renal ischemia/reperfusion is a major cause of acute kidney injury. However, the pathogenic mechanisms underlying renal ischemia/reperfusion injury (IRI) are not fully defined. Here, we investigated the role of PTEN, a dual protein/lipid phosphatase, in the development of ischemic AKI in mice. Pharmacological inhibition of PTEN with bpV(HOpic) exacerbated renal dysfunction and promoted tubular damage in mice with IRI compared with vehicle-treated mice with IRI. PTEN inhibition enhanced tubular cell apoptosis in kidneys with IRI, which was associated with excessive caspase-3 activation. Furthermore, PTEN inhibition expanded the infiltration of neutrophils and macrophages into kidneys with IRI, which was accompanied by increased expression of the proinflammatory molecules. These results have demonstrated that PTEN plays a crucial role in the pathogenesis of ischemic acute kidney injury through regulating tubular cell apoptosis and inflammation suggesting PTEN could be a potential therapeutic target for acute kidney injury.

## Introduction

Acute kidney injury (AKI) is a common clinical condition associated with increased mortality and morbidity, prolonged hospital stay, and accelerated chronic kidney disease (CKD)^1, 2^. Ischemia-reperfusion injury (IRI) is a major cause of AKI^1, 3^. However, the pathogenesis of ischemic AKI is complex and incompletely understood. The pathophysiology of ischemic AKI includes hemodynamic alteration, inflammation, endothelial dysfunction, and epithelial cell injury which eventually result in acute tubular damage and renal dysfunction^1, 4, 5^. Currently, there is no effective therapy for ischemic AKI. Therefore, a better understanding of the pathogenic mechanisms underlying IRI is essential to develop effective therapy for ischemic AKI.

Phosphatase and tensin homologue deleted from chromosome 10 (PTEN) is a dual protein and phospholipid phosphatase^6^. The functional role of PTEN includes the regulation of cell growth, adhesion, and migration^7, 8^. The primary function of PTEN acts as a lipid phosphatase that converts phosphatidylinositol (3,4,5)-triphosphate (PIP3) to phosphatidylinositol (4,5)-triphosphate (PIP2) thereby downregulating phosphatidylinositol 3 kinase (PI3K)/Akt signaling^6, 9, 10^. Recent studies have shown that PTEN regulates kidney morphology, podocyte injury and renal fibrosis. PTEN loss is associated with increased transforming growth factor (TGF)-β signaling and renal fibrosis^11^. Mice with proximal tubule deletion of PTEN develop larger kidneys characterized by proximal tubule cell hypertrophy^12^. We have recently demonstrated that loss of PTEN in podocytes aggravates diabetic nephropathy through regulation of cytoskeletal rearrangement^13^. However, the functional role of PTEN in ischemic AKI is not known.

In this study, we examined the effect of pharmacological inhibition of PTEN on the pathogenesis of AKI in a mouse model of ischemia-reperfusion injury. Our results show that pharmacological inhibition of PTEN with bpV(HOpic), a selective PTEN inhibitor^13^, exacerbates ischemic AKI by promoting apoptosis and inflammation.

## Results

### PTEN is activated in the kidney in response to ischemic AKI

**Figure 1:**
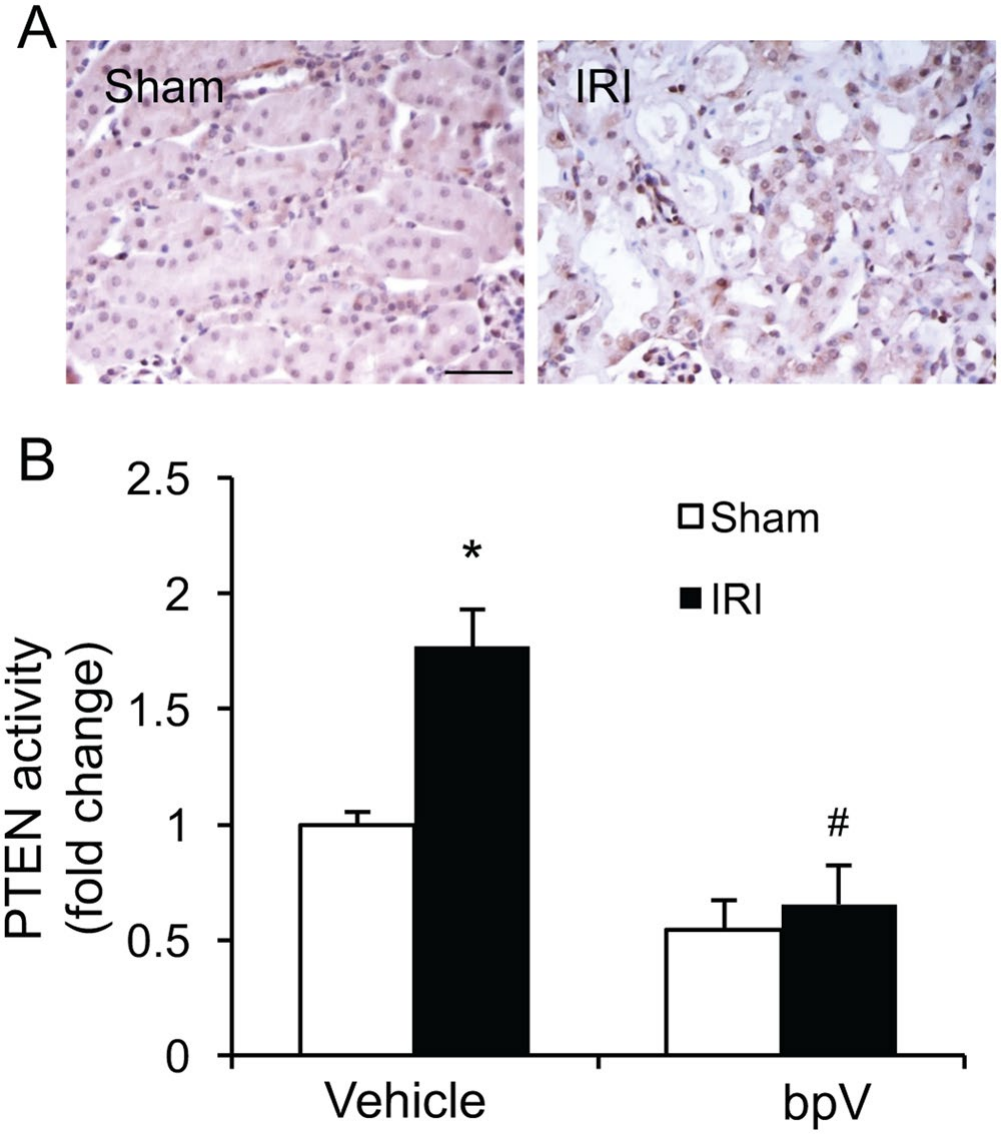
PTEN is activated in the kidney with IRI. (**A**) Representative photomicrographs of kidney sections stained with PTEN (brown) and counterstained with hematoxylin (blue). Scale bar, 50 μm. (**B**) PTEN activity was measured in kidney homogenates and expressed as fold changes. *P < 0.05 vs. Vehicle Sham, ^#^ *P* < 0.05 vs. vehicle IRI. n = 6 per group.

We first determined whether PTEN is induced in the kidney in a mouse model of AKI induced by 30 min of ischemia followed by 24 hr of reperfusion. Sham-operated mice were served as controls. Kidney sections were stained for PTEN. Immunohistochemical staining revealed that PTEN is expressed at a low level in the kidney of sham-operated mice, which was markedly induced in the interstitial cells of the kidney following ischemic AKI (Fig. 1A). We next examined if IRI affects PTEN activity in the kidney using the malachite green phosphatase assay^14^. The results showed that PTEN activity increased in the kidney following ischemic AKI, which was significantly inhibited by bpV(HOpic) (Fig. 1B). These data indicate that PTEN may regulate the pathogenesis of ischemic AKI.

### PTEN inhibition exacerbates kidney dysfunction following IRI

**Figure 2:**
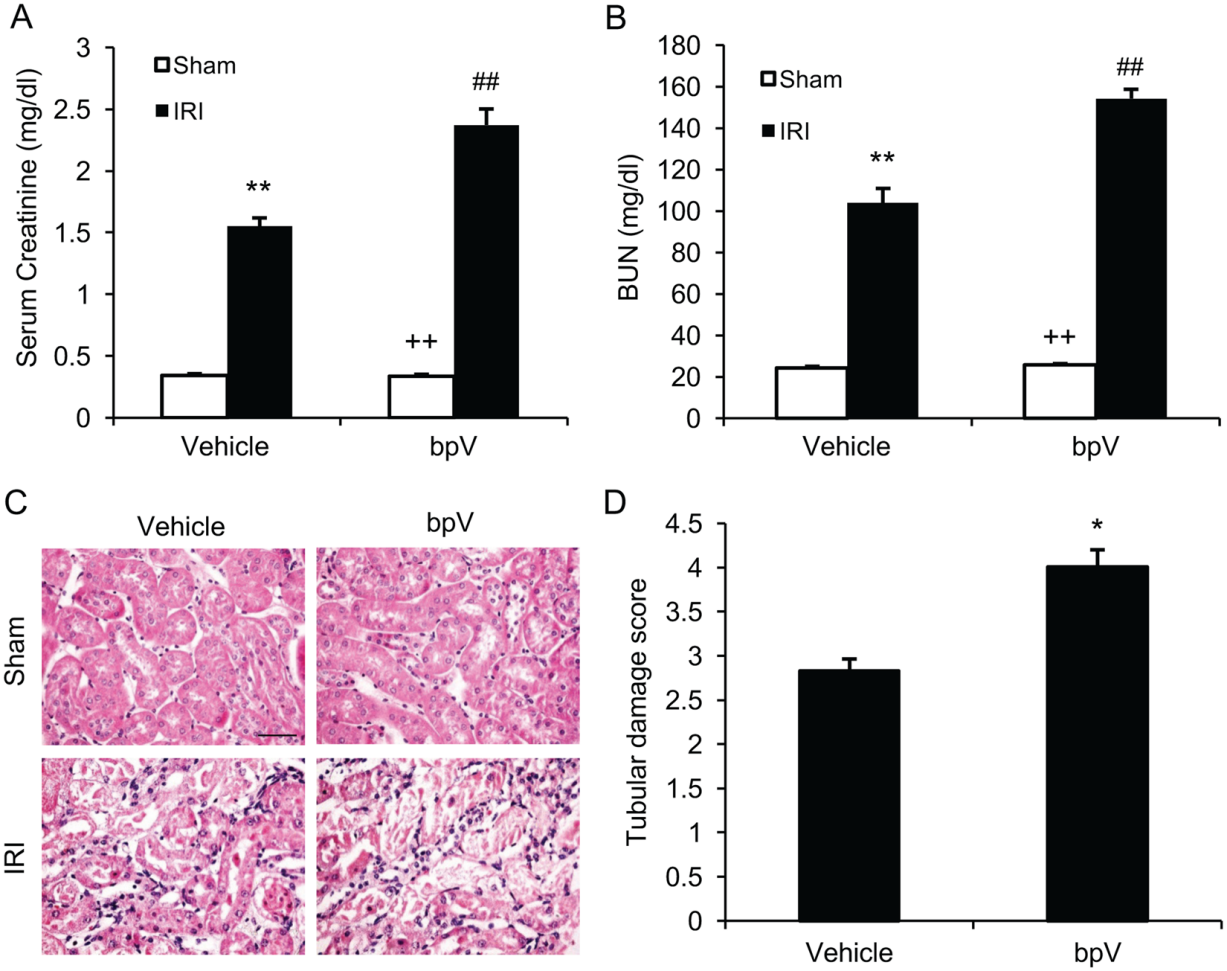
PTEN inhibition exacerbates kidney injury after IRI. (**A**) Serum creatinine increased markedly in PTEN inhibitor bpV treated mice with IRI. **P < 0.01 vs. Vehicle Sham, ^++^ *P* < 0.01 vs. bpV IRI, ^##^ *P* < 0.01 vs. Vehicle IRI. n = 6 per group. (**B**) Blood serum urea nitrogen (BUN) increased significantly in bpV treated mice with IRI. **P < 0.01 vs. Vehicle Sham, ^++^ *P* < 0.01 vs. bpV IRI, ^##^ *P* < 0.01 vs. Vehicle IRI. n = 6 per group. (**C**) Representative photomicrographs of kidney sections stained with hematoxylin and eosin. Scale bar, 50 μm. (**D**) Quantitative analysis of tubular damage after IRI. *P < 0.05 vs. Vehicle IRI. n = 6 per group.

To determine the role of PTEN in the pathogenesis of ischemic AKI, mice were subjected to 30 min of ischemia, followed by 24 hr of reperfusion. Sham-operated mice were served as controls. PTEN inhibitor, bpV(HOpic) (200 μg/kg), or vehicle was intraperitoneally administered 1 h before ischemia and then administered every 6 h after ischemia for 24 hr. Renal IRI led to significant kidney dysfunction with an increase in serum creatinine and BUN (Fig. 1A and B). In contrast, kidney dysfunction was even worsened in bpV(HOpic) treated mice with much higher elevation of serum creatinine and BUN (Fig. 2A and B). Consistent with the worsening of kidney dysfunction in bpV(HOpic) treated mice with IRI, there was a much greater increase in histological injury of the kidney as reflected by more tubular injury, tubular dilation, and intratubular cast formation in bpV(HOpic) treated mice with IRI compared with vehicle-treated mice with IRI (Fig. 2C and D). These data indicate that PTEN inhibition exacerbates ischemic AKI.

### PTEN inhibition enhances apoptotic cell death in the kidney with IRI

**Figure 3:**
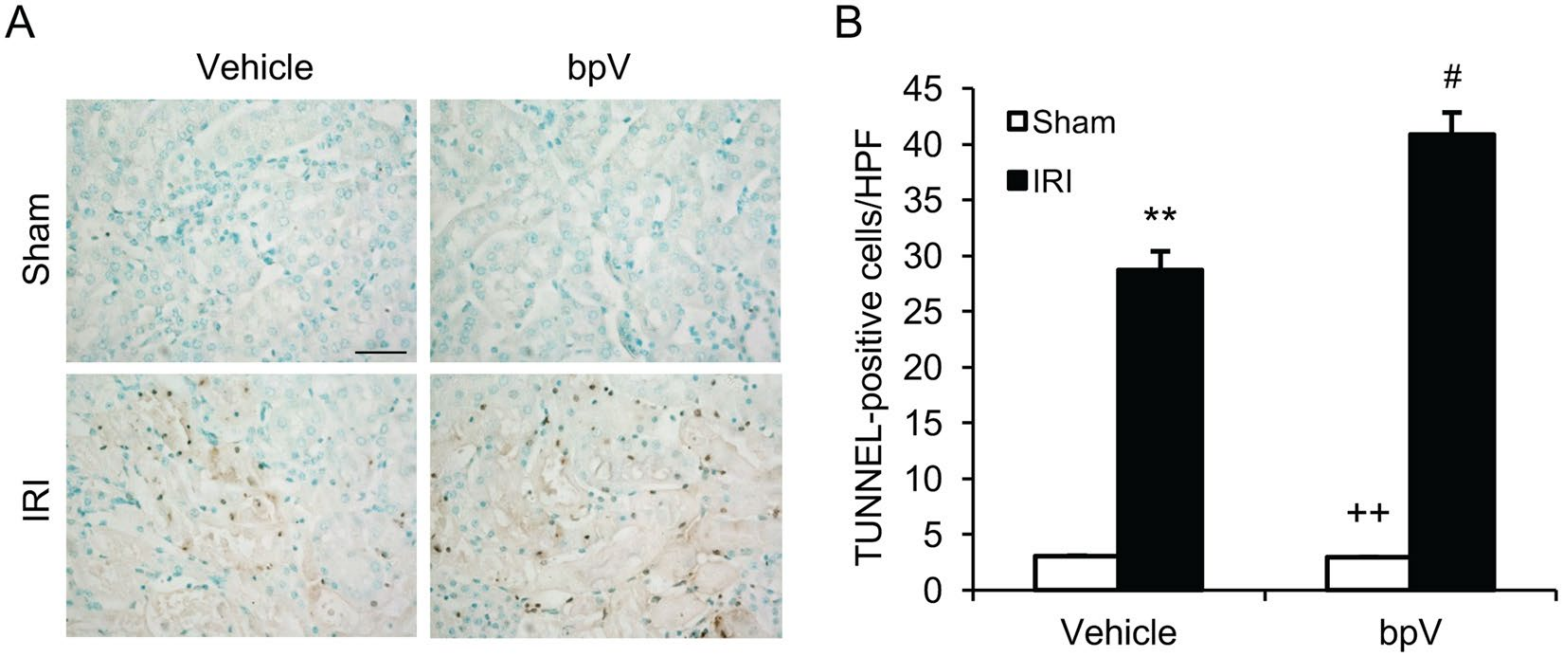
PTEN inhibition promotes tubular epithelial cell apoptosis in IRI kidney. (**A**) Representative photomicrographs of kidney sections stained for apoptotic cells (brown) and counterstained with methyl green (green) in kidneys. Scale bar, 50 μm. (**B**) Quantitative analysis of TUNEL-positive cells in the kidneys. **P < 0.01 vs. Vehicle Sham, ^++^ *P* < 0.01 vs. bpV IRI, ^#^ *P* < 0.05 vs. Vehicle IRI. n = 6 per group. HPF, high power field; TUNEL, terminal transferase dUTP nick-end labeling.

Tubular epithelial cell apoptosis plays an important role in the pathogenesis of ischemic kidney injury^1, 15, 16^. Therefore, we determine the effect of PTEN inhibition on tubular epithelial cell apoptosis following IRI. Kidney IRI significantly increased the number of tubular apoptotic cells in the kidneys as assessed by TUNEL staining; whereas bpV(HOpic) treatment further increased the number of tubular apoptotic cells in IRI kidneys (Fig. 3A and B). These data indicate that inhibition of PTEN promotes tubular epithelial cell apoptosis.

**Figure 4:**
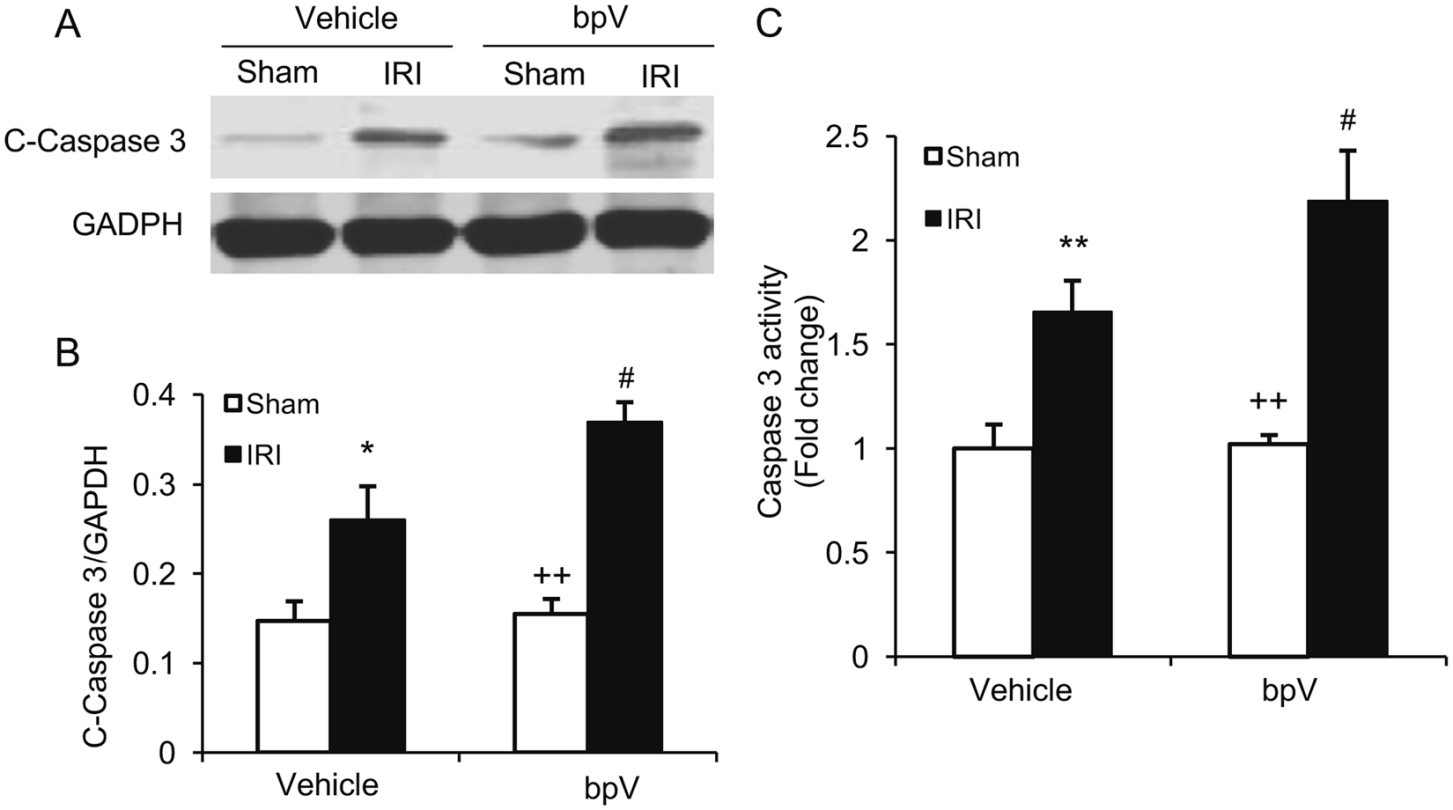
PTEN inhibition increases caspase 3 activation in IRI kidney. (**A**) Representative western blots show cleaved caspase 3 protein levels in the kidneys after sham or IRI. (**B**) Quantitative analysis of cleaved caspase 3 protein levels in the kidneys. *P < 0.05 vs. Vehicle Sham, ^++^ *P* < 0.01 vs. bpV IRI, ^#^ *P* < 0.05 vs. Vehicle IRI. n = 6 per group. (**C**) Caspase 3 activity is increased in bpV treated IRI kidney than vehicle treated IRI kidney. **P < 0.01 vs. Vehicle Sham, ^++^ *P* < 0.01 vs. bpV IRI, ^#^ *P* < 0.05 vs. vehicle IRI. n = 6 per group.

Caspase 3 is the final effector in apoptotic cell death^17^. We then determined the effect of PTEN inhibition on caspase 3 activation in the kidney following IRI. Western blot analysis showed that the levels of cleaved caspase 3 in the kidneys were significantly higher in bpV(HOpic)-treated mice after IRI than vehicle-treated mice with IRI (Fig. 4A). Furthermore, caspase 3 activity assay revealed that caspase 3 activity was significantly increased in bpV(HOpic)-treated kidney with IRI compared with vehicle-treated kidney with IRI (Fig. 4B). These data indicate that PTEN inhibition enhances caspase 3 activation in the kidney after IRI.

### PTEN inhibition augments inflammatory cell infiltration in IRI kidney

**Figure 5:**
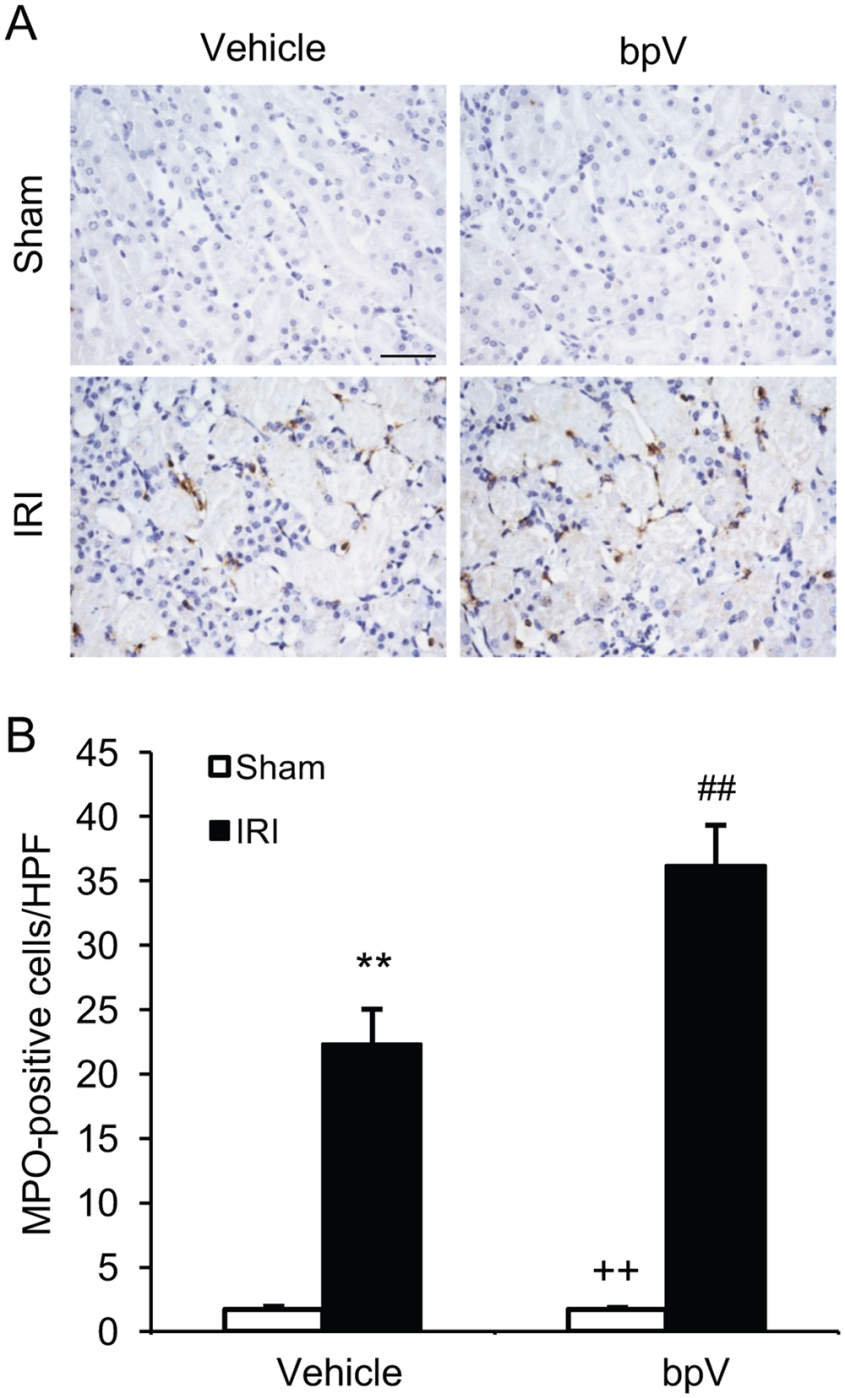
PTEN inhibition promotes neutrophils infiltration into IRI kidney. (**A**) Representative photomicrographs of kidney sections stained for MPO (a neutrophils marker) (brown) and counterstained with hematoxylin (blue). Scale bar, 50 μm. (**B**) Quantitative analysis of neutrophils in the kidneys. **P < 0.01 vs. Vehicle Sham, ^++^ *P* < 0.01 vs. bpV IRI, ^##^ *P* < 0.01 vs. Vehicle IRI. n = 6 per group.

**Figure 6:**
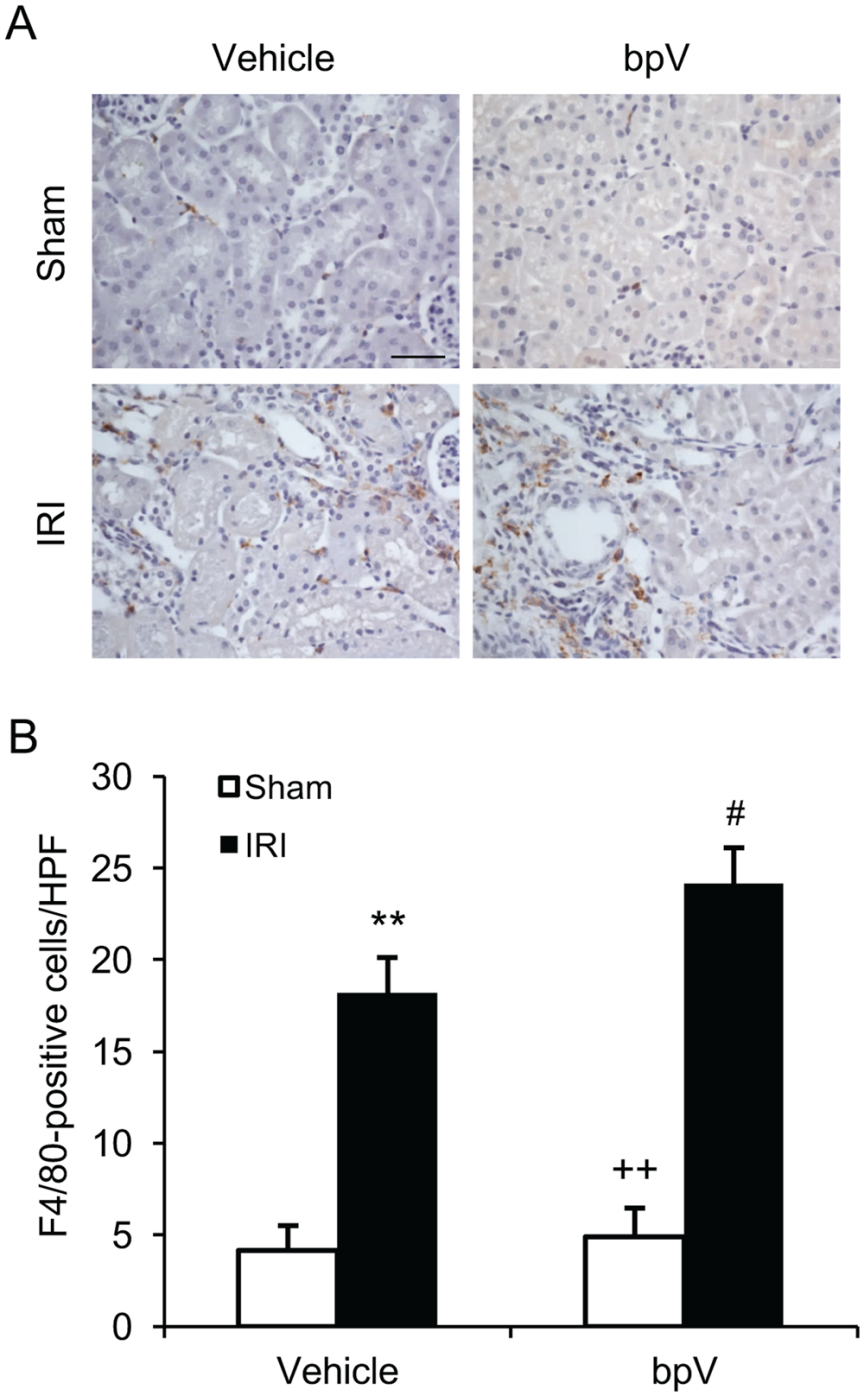
PTEN inhibition enhances macrophage infiltration into IRI kidney. (**A**) Representative photomicrographs of kidney sections stained for F4/80 (a macrophage marker) (brown) and counterstained with hematoxylin (blue). Scale bar, 50 μm. (**B**) Quantitative analysis of macrophages in the kidneys. **P < 0.01 vs. Vehicle Sham, ^++^ *P* < 0.01 vs. bpV IRI, ^#^ *P* < 0.05 vs. Vehicle IRI. n = 6 per group.

Inflammatory cells have been shown to infiltrate the kidney during the injury phase and participate in the pathogenesis of renal IRI^18^. We next investigated whether PTEN inhibition affects inflammatory cell infiltration into the kidney in response to IRI. Kidney sections were stained with antibodies against MPO, a neutrophil marker, and F4/80, a macrophage marker. The number of neutrophils were increased significantly in the IRI kidneys of mice treated with vehicle, which were further increased in the IRI kidneys of mice treated with bpV(HOpic) (Fig. 5). Similarly, bpV(HOpic) treatment resulted in a significant increase in macrophages in the kidneys of mice with IRI compared with vehicle-treated mice with IRI (Fig. 6). These data suggest that PTEN inhibition promotes inflammatory cell infiltration into the kidney during IRI.

### PTEN inhibition upregulates inflammatory mediators in IRI kidney

**Figure 7:**
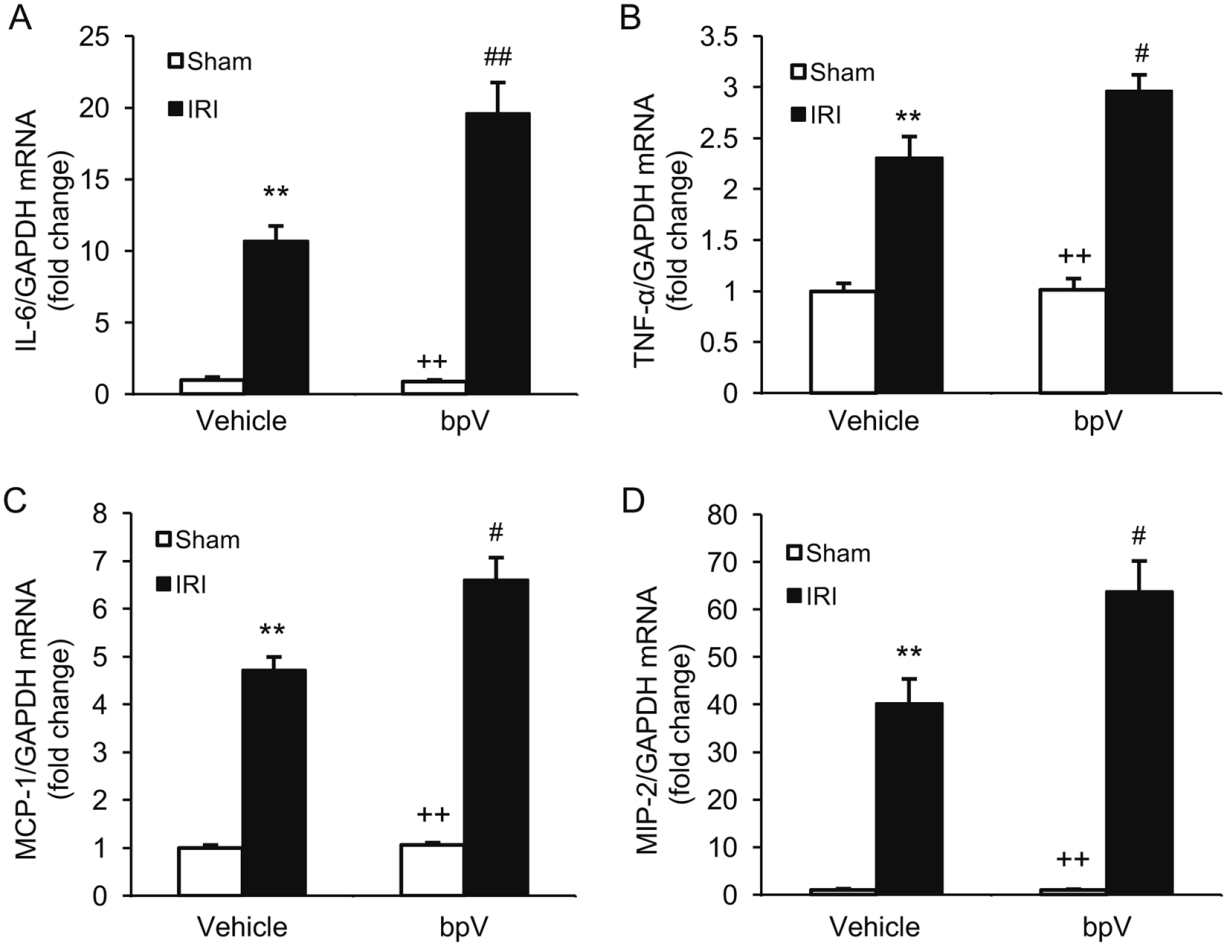
PTEN inhibition increases gene expression of inflammatory molecules in the kidneys with IRI. (**A**) IL-6 mRNA expression is increased in bpV-treated IRI kidneys compared with vehicle-treated IRI kidneys. **P < 0.01 vs. Vehicle Sham; ^++^ *P* < 0.01 vs. bpV IRI; ^##^ *P* < 0.01 vs. Vehicle IRI. n = 4 per group. (**B**) TNF-α mRNA expression is enhanced in bpV-treated IRI kidneys compared with vehicle-treated IRI kidneys. **P < 0.01 vs. Vehicle Sham; ^++^ *P* < 0.01 vs. bpV IRI; #*P* < 0.05 vs. Vehicle IRI. n = 4 per group. (**C**) MCP-1 mRNA expression is increased in bpV-treated IRI kidneys compared with vehicle-treated IRI kidneys. **P < 0.01 vs. Vehicle Sham; ^++^ *P* < 0.01 vs. bpV IRI; ^#^ *P* < 0.05 vs. Vehicle IRI. n = 4 per group. (**D**) MIP-2 mRNA expression is augmented in bpV-treated IRI kidneys compared with vehicle-treated IRI kidneys. **P < 0.001 vs. Vehicle Sham; ^++^ *P* < 0.001 vs. bpV IRI; ^#^ *P* < 0.05 vs. Vehicle IRI. n = 4 per group.

Inflammatory cytokines and chemokines are crucial mediators in the pathogenesis of ischemic AKI^16, 18^. We then examined the effect of PTEN inhibition on the expression of inflammatory mediators in the kidney by real-time RT-PCR. The mRNA levels of interleukin 6 (IL-6), tumor necrosis factor α (TNF-α), MCP-1 and MIP-2 were increased significantly in IRI kidneys of mice treated with vehicle compared with vehicle-treated sham–operated controls (Fig. 7). Inhibition of PTEN with bpV(HOpic) further increased the expression levels of these proinflammatory molecules in the IRI kidneys (Fig. 7). These results suggest that PTEN inhibition augments inflammatory chemokine and cytokine expression in the kidney during IRI injury.

## Discussion

PTEN has shown to regulate cell growth, adhesion, and migration^7, 8^. However, its role in ischemic AKI is not known. In the present study, PTEN inhibition with bpV(HOpic) exacerbates kidney dysfunction through augmenting inflammatory cell infiltration and proinflammatory molecule production. Our results identify PTEN as a critical regulator of the pathogenesis of renal IRI. These results suggest that PTEN plays an important role in the pathogenesis of renal IRI through the regulation of inflammation and apoptosis.

PTEN is first identified as a tumor-suppressor gene located on chromosome 10, which is mutated in a large number of tumors^19^. The crystal structure of PTEN reveals that PTEN contains a phosphatase domain that is similar to protein phosphatases but also has an enlarged active site important for the accommodation of the phosphoinositide substrate^20^. The principal substrate of PTEN is phosphatidylinositol (3,4,5)-trisphosphate (PIP3) *in vitro* ^10, 20^. PIP3 is a lipid second messenger generated by the activation of phosphoinositide 3-kinase (PI3K)^20^. Thus, PTEN negatively regulated PI3K signaling by dephosphorylating PIP3 and downregulating its downstream effector including AKt/PKB kinase which has potent pro-migration and growth stimulatory effect^20^. A function role of PTEN phosphatase activity in tumor suppressor has been extensively studied^21, 22^. Recent evidence indicates that PTEN is involved in kidney development and pathogenesis of kidney disease. For example, one study shows that PTEN loss is associated with increased TGF-β signaling and renal fibrosis^11^. Another study reports that mice with proximal tubule deletion of PTEN develop larger kidneys characterized by proximal tubule cell hypertrophy^12^. We have recently demonstrated that loss of PTEN in podocytes aggravates diabetic nephropathy through regulation of cytoskeletal rearrangement^13^. However, the functional role of PTEN in ischemic AKI is unknown. In the present study, our results have showed that inhibition of PTEN with bpV(HOpic) augments renal dysfunction and promotes tubular damage in mice with IRI compared with vehicle-treated mice with IRI. These results indicate that PTEN inhibition promotes kidney dysfunction in response to IRI.

Apoptosis of tubular epithelial cells contributes to the development of ischemic AKI^15, 16^. In the present study, IRI resulted in TUNEL-positive apoptotic cell death in the kidney, which is further increased in mice treated with bpV(HOpic). The Caspase family of cysteine proteases mediate the initiation and execution of the mammalian apoptotic cell death program. Caspase-3 is activated by death ligand and mitochondrial pathways. Caspase-3 activation is a key mechanism underlying the pathogenesis of IRI-induced apoptotic cell death in tubular epithelial cells^23^. In the present study, we have examined the effect of PTEN inhibition with bpV(HOpic) on IRI-induced caspase-3 activation in the kidney. Our results have demonstrated that caspase-3 is significantly activated in the kidney following IRI, whereas bpV(HOpic) treatment leads to greater caspase-3 activation in the kidney with IRI. These data suggest that PTEN inhibits caspase-3 activation and tubular epithelial cell apoptosis in response to IRI.

In response to kidney ischemia-reperfusion injury, innate and adaptive immune cells are recruited into the kidney participating in the pathogenesis of ischemic AKI^1, 16, 18^. In the present study, we have shown that neutrophils and macrophages infiltrate into the kidney following IRI. PTEN inhibition increases infiltration of neutrophils and macrophages into the kidney with IRI. These data indicate that PTEN inhibition promotes renal IRI through increasing the infiltration of neutrophils and macrophages into the kidney.

Inflammatory cytokines and chemokines are crucial mediators in the pathogenesis of ischemic AKI^16, 18^. Recent studies have shown that proinflammatory cytokines and chemokines such as TNF-α, IL-6, MCP-1, and MIP-2 contribute to the development of renal IRI. In the present study, we have shown that inflammation molecules such as IL-6, TNF-α, MCP-1 and MIP-2 are elevated in IRI kidney. Inhibition of PTEN with bpV(HOpic) considerably enhances the production of these inflammatory molecules. These results indicate PTEN affects IRI through regulating the inflammatory cells infiltration and inflammatory cytokines production.

In summary, our study shows that PTEN is involved in regulating the pathogenesis of renal IRI. Pharmacological inhibition of PTEN promotes accumulation of circulating inflammatory cells into the kidney and production of inflammatory molecules, which exacerbates the development of ischemic AKI. These data suggest that PTEN activation could be a potential therapeutic approach for ischemic AKI.

## Methods

### Animals

The animal experiments were conducted according to the Guide for the Care and Use of Laboratory Animals and were approved by the Institutional Animal Care and Use Committee of Baylor College of Medicine. Wild type (WT) C57BL/6 mice were purchased from the Jackson Laboratory (Bar Harbor, ME). Male WT mice, 8 to 12 weeks old, weighing 20 to 30 grams, were anesthetized by intraperitoneal injection of ketamine (80 mg/kg) and xylazine (10 mg/kg). Kidneys were exposed through flank incision and were subjected to ischemia by clamping renal pedicles with non-traumatic microaneurism clamps. After 30 minutes, the clamps were removed and blood reflow was confirmed. Body temperature was maintained at 36.5–37.5 °C throughout the procedure. Sham control mice underwent identical surgical procedure but without pedicle clamping. Animals were sacrificed at 24 hours after reperfusion. Kidneys were perfused and harvested^16^.

### PTEN Phosphatase Activity Measurement

Kidney tissues were homogenized in ice-cold lysis buffer (25 mM Tris PH 8.0, 150 mM NaCl, 1%NP-40, 1 mM EDTA, 5% Glycerol and protease inhibitor cocktail). Tissue homogenates were centrifuged at 14000 × g for 10 minutes at 4 °C. The pellets were incubated with PTEN antibody and protein A agarose beads overnight at 4 °C. The beads were washed with PTEN reaction buffer (25 mM Tris-Cl, PH 7.4, 140 mM NaCl, 2.7 mM KCl with 10 mM DTT). After washing, the beads were resuspended in PTEN reaction buffer and PTEN enzyme reaction was initiated by adding PI(3,4,5)P3 substrate for 1 hour at 37 °C. The enzyme reaction was terminated by adding Malachite Green solution for 20 minutes at room temperature. PTEN phosphatase activity was measured at 620 nm using a plate reader.

### Renal Function Measurement

Serum creatinine was measured using a creatinine assay kit (BioAssay Systems, Hayward, CA) and blood urea nitrogen was determined fluorometrically as described^16, 24, 25^.

### Renal Morphology

Kidney tissues were fixed in 10% buffered formalin, embedded in paraffin, and cut at 4 µm thickness. After deparaffinization and rehydration, kidney sections were stained with hematoxylin and eosin. Tissue damage was examined in a blinded manner and scored according to the percentage of damaged tubules: 0, no damage; 1, less than 25% damage; 2, 25–50% damage; 3, 50–75% damage; and 4, more than 75% damage as reported^16, 26^.

### Immunohistochemistry

Immunohistochemical staining was performed on paraffin sections. Antigen retrieval was performed with antigen unmasking solution (Vector Laboratories, Burlingame, CA). Endogenous peroxidase activity was quenched with 3% H_2_O_2_. After blocked with 5% normal serum, slides were incubated with primary antibodies in a humidified chamber overnight. After washing, slides were incubated with appropriate secondary antibodies and ABC solution sequentially per ABC Elite kit (Vector Laboratories, Burlingame, CA). Immunoreactivity was revealed by incubation in DAB solution. Nuclear staining was performed with hematoxylin. The slides were dehydrated, cleared, and mounted. The images were acquired and analyzed by NIS Element software with Nikon microscope image system^16^.

### Caspase 3 Activity Assay

Caspase 3 Activity was measured using Caspase-3 Colorimetric Activity Assay Kit (Millipore, Billerica, MA). Briefly, kidney tissue was homogenized in chilled cell lysis buffer and incubated on ice for 10 minutes. After centrifugation for 1 min at 10,000 × g, reaction buffer containing 10 mM DTT and DEVD-pNA substrate were added to the supernatant and incubated for 1 hour at 37 °C. Samples were read at 405 nm in a microtiter plate reader. Caspase 3 activity was determined by comparing with the level of the control.

### Apoptosis Detection

TUNEL assay was performed to evaluate apoptosis using ApopTag^R^ plus Peroxidase *in Situ* Apoptosis Detection Kit (Millipore, Billerica, MA) according to manufacturer’s instruction. The number of TUNEL-positive cells per high-power field were counted and analyzed in a blinded fashion^16, 27^.

### Quantitative Real-Time RT-PCR

Total RNA was extracted from frozen kidney tissues with TRIzol reagent (Invitrogen, Carlsbad, CA) followed by RNase-free DNase I (Roche, Madison, WI). Aliquots (1 μg) of total RNA were reverse transcribed using SuperScript II reverse transcriptase. Real-time PCR was performed using IQ SYBR green supermix reagent (Bio-Rad, Hercules, CA) with a Bio-Rad real-time PCR machine^28–30^. The expression levels of the target genes were normalized to GAPDH level in each sample. The primer sequences were: IL-6 - forward, 5′-AGGATACCACTCCCAACAGACCTG-3′, reverse, 5′-CTGCAAGTGCATCATCGTTGTTCA-3′; TNF-α - forward, 5′-CATGAGCACAGAAAGCATGATCCG-3′ reverse, 5′-AAGCAGGAATGAGAAGAGGCTGAG-3′; MCP-1 - forward, 5′-TCACCTGCTGCTACTCATTCACCA-3′, reverse, 5′-TACAGCTTCTTTGGGACACCTGCT-3′; MIP-2 - forward, 5′-AAAGTTTGCCTTGACCCTGAAGCC-3′, reverse, 5′-TCCAGGTCAGTTAGCCTTGCCTTT-3′; GAPDH - forward, 5′-CCAATGTGTCCGTCGCGTGGATCT-3′, reverse, 5′-GTTGAAGTCGCAGGAGACAACC-3′.

### Western Blot Analysis

Proteins were extracted using RIPA buffer containing cocktail proteinase inhibitors and quantified with a Bio-Rad protein assay. Equal amounts of protein (50 μg) were separated on SDS-polycrylamide gels and then transferred onto nitrocellulose membranes. The membranes were incubated with primary antibodies overnight followed by incubation with appropriate fluorescence-conjugated secondary antibodies. The proteins of interest were analyzed using an Odyssey IR scanner, and signal intensities were quantified using NIH Image/J software^28–35^.

### Statistical Analysis

All data were expressed as mean ± SEM. Multiple group comparisons were performed by ANOVA followed by the Bonferroni procedure for comparison of means. Comparisons between two groups were analyzed by the two-tailed *t* test. *P* < 0.05 was considered statistically significant.
